# Comparison of Ligation-Mediated PCR Methods in Differentiation of *Mycobacterium tuberculosis* Strains

**DOI:** 10.1155/2014/782071

**Published:** 2014-02-16

**Authors:** Anna Zaczek, Anna Brzostek, Arkadiusz Wojtasik, Anna Sajduda, Jaroslaw Dziadek

**Affiliations:** ^1^Department of Biochemistry and Cell Biology, University of Rzeszow, 35-601 Rzeszow, Poland; ^2^Institute of Medical Biology, Polish Academy of Science, 93-232 Lodz, Poland; ^3^Department of Microbial Genetics, Faculty of Biology and Environmental Protection, University of Lodz, 90-237 Lodz, Poland

## Abstract

Fast and inexpensive identification of epidemiological links between limited number of *Mycobacterium tuberculosis* strains is required to initially evaluate hospital outbreaks, laboratory crosscontaminations, and family or small community transmissions. The ligation-mediated PCR methods (LM-PCR) appear sufficiently discriminative and reproducible to be considered as a good candidate for such initial, epidemiological analysis. Here, we compared the discriminative power of the recently developed in our laboratory fast ligation amplification polymorphism (FLAP) method with fast ligation-mediated PCR (FLiP). Verification of the results was based on analyzing a set of reference strains and RFLP-IS*6110* typing. The HGDI value was very similar for both LM-PCR methods and RFLP-IS*6110* typing. However, only 52% of strains were correspondingly grouped by both FLiP and FLAP methods. Differentiation by FLAP method demonstrated a limited similarity to IS*6110*-RFLP (37,7%). As much as 78,7% of strains were grouped identically when differentiated by FLiP and IS*6110*-RFLP methods. The analysis differentiated 31, 35, and 36 groups when using FLAP, FLiP, and RFLP-IS*6110* methods, respectively.

## 1. Introduction

Recent development of molecular methods has substantially improved the identification of many bacterial pathogens, both at the species and strain levels. *M. tuberculosis*, the causative agent of tuberculosis, is still one of the most dangerous human pathogens causing high morbidity and mortality worldwide. The genetic typing of mycobacteria has greatly improved knowledge about tuberculosis epidemiology and enabled a molecular-guided control of the disease. Various genetic markers are used in molecular epidemiology of tuberculosis. In particular, identification of repeated sequences in mycobacterial genome and their analysis at molecular level allowed to develop the intraspecies discrimination methods for mycobacteria [1].

The current international standard for epidemiological typing of *M. tuberculosis *is restriction fragment length polymorphism (RFLP) based on the detection of variability in the number of copies and chromosomal locations of IS*6110* insertion sequences [2–5]. The second widely used method is mycobacterial interspersed repetitive unit-variable number of tandem repeats typing (MIRU-VNTR) based on variable number of tandem repeats [6]. Finally spoligotyping (spacer oligonucleotide typing) based on polymorphism in the chromosomal direct repeat (DR) locus is often used as a fast screening method [7]. Interesting alternative for the methods mentioned above are those based on ligation-mediated PCR (LM-PCR), which have proven useful in epidemiological analysis of a number of bacterial species [8–10]. Such methods can be adapted to mycobacterial typing when they are based on variability in IS*6110* flanking regions [11–15].

Here, we assess the usefulness of a recently described in our group LM-PCR method, termed fast ligation amplification polymorphism (FLAP), for differentiation of *M. tuberculosis* strains [15]. We present the results of its application in context of published results of reference set [14, 16] and compare its discriminatory power to that of IS*6110*-RFLP and FLiP (fast ligation-mediated PCR) methods.

## 2. Materials and Methods

### 2.1. Bacterial Strains

The 61 strains used in this analysis were obtained in 2006-2007 from patients hospitalized in the Center for Lung Diseases Treatment and Rehabilitation in Lodz, Poland. All strains were tested for susceptibility to isoniazid, rifampicin, pyrazinamide, streptomycin, and ethambutol using the Bactec 460 TB system (BD Diagnostic Systems, Sparks, MD, USA), as described previously [16]. This set of strains was previously characterized by IS*6110*-RFLP analysis, 15 locus MIRU-VNTR typing, and spoligotyping [16].

### 2.2. DNA Preparation

Genomic DNA was extracted and purified from all the isolates using the protocol by van Embden et al. [2]. The concentration of DNA was measured with NanoDrop ND-1000 spectrophotometer (NanoDrop Technologies, Montchanin, DE, USA).

### 2.3. The FLiP Method

The FLiP analysis was performed as originally described by Reisig et al. [13]. Briefly, the method is based on the ligation of oligonucleotide adaptors. Following restriction digestion, genomic DNA is ligated with an adapter composed of two oligonucleotides, one of which is complementary to the end created by restrictase, while the other contains uracil instead of thymine. A pair of starters is used for amplification; one of them is specific to the IS*6110* sequence and the other is complementary to the oligonucleotide ligated with restricted genomic DNA fragments. Amplification products are analyzed using electrophoresis; the obtained band patterns are strain specific.

### 2.4. The FLAP Method

The FLAP method was performed as we previously described [15]. Briefly, genomic DNA of *M. tuberculosis* strains was digested with *Pvu*II and *Sal*I restriction enzymes. The *Pvu*II endonuclease recognizes a single nucleotide sequence within IS*6110* and generates blunt ends. After the digestion step, oligonucleotide adaptors (36 and 40 nucleotides in length) are ligated to *Sal*I cohesive ends. All restriction fragments are used as templates for PCR amplification, with one primer complementary to adaptor sequence and the second primer complementary to the inner fragment of IS*6110*. The PCR products were separated on acrylamide gels and visualized by UV light illumination to generate the FLAP patterns.

### 2.5. Clustering and Computer Analysis

The fingerprint patterns obtained by both methods were analysed by using BioNumerics software, version 5.0 (Applied Maths, Sint-Martens-Latem, Belgium). Dendrograms were generated based on the Unweighted Pair Group Method with Arithmetic Averages (UPGMA) algorithm for clustering and Dice similarity coefficient. The Hunter-Gaston discriminatory index (HGDI) was calculated as previously described and used to evaluate the discriminatory power of the typing methods [17].

## 3. Results and Discussion

The “gold standard” method for epidemiological typing of *M. tuberculosis *is IS*6110*-RFLP analysis providing the best resolution at the population level [2–5]. It seems obvious that appropriate epidemiological analyses of *M. tuberculosis *clinical strains should be based on more than one molecular method. Recently developed PCR-based genotyping methods are rapid, do not require a large quantity of purified DNA, and provide reproducible digital results. In particular, the relatively novel ligation-mediated PCR FLiP and FLAP typing methods seem to be promising alternatives for genotyping of *M. tuberculosis*, as well as for the detection of genotypic heterogeneity, mixed infection, and crosscontamination of mycobacterial samples [11–15].

In this study, we compared the results obtained by FLAP for 61 strains of *M. tuberculosis*, isolated from TB patients in 2006-2007, with both FLiP and IS*6110* RFLP methods, and estimated their discriminatory power by HGDI. All the methods are based on detection of insertion sequence IS*6110*.

The FLAP analysis subdivided the 61 analyzed strains into 31 clusters; 13 of which demonstrated unique patterns. The remaining 48 strains were grouped into two clusters of 5 strains each, three clusters of 4 strains each, and thirteen clusters of 2 strains each (Table 1).

The total of 35 FLiP patterns were detected and distributed in 14 clusters within 40 strains (65,6%) and 21 unique patterns (34,4%). One cluster consisted of 8 strains with identical FLiP pattern, three clusters contained 5, 4, and 3 strains, respectively, while eleven clusters comprised 2 strains each (Table 1).

The discriminatory power of the FLAP typing for the 61 *M. tuberculosis *isolates, calculated as HGDI, was 0.9757 compared to 0.9713 for FLiP method.

Previously performed analysis by reference methods, IS*6110*-RFLP and MIRU-VNTR typing, grouped this set of strains into 36, for IS*6110*-RFLP, and 27 patterns, for MIRU-VNTR, and gave the resolving power, 0.9743 and 0.9697, respectively [16].

We observed differences in the number of copies of IS*6110* when determined by reference method IS*6110 *RFLP and LM-PCR methods (FLiP and FLAP). Therefore minimal and maximal number of bands in particular patterns were calculated. The copy number of IS*6110*, as determined by the reference method in each of 61 strains ranged from 6 to 14. The majority, 48 (78,7%) of the strains, contained 8–12 copies. FLAP patterns obtained for the same set of strains varied from 4 to 12, with 51 (83,6%) of strains containing 7–9 bands in pattern. In contrast, the number of IS*6110* in FLiP patterns varied from 3 to 8, and 49 (80,3%) of strains possessed 6–8 bands in pattern.

We found that FLAP patterns of 46 strains (75,4%) possessed more bands in particular than when analyzed by FLiP, 5 strains (8,2%) consisted of less bands, and 10 (16,4%) of strains contained the same number of bands in both methods. Next, we compared band patterns between FLAP and IS*6110 *RFLP methods. 41 strains (67,2%) possessed less bands than when analyzed by IS*6110 *RFLP typing; in eight patterns (13,1%) we observed more bands while 12 (19,7%) strains contained identical number of band patterns. Comparison of patterns obtained by FLiP and IS*6110 *RFLP typing revealed lower number of bands in 56 strains (91,8%) analyzed by FLIP when compared to IS*6110 *RFLP, and 2 strains (3,3%) possessed higher number of bands, and 3 (4,9%) strains presented identical number of bands in these two methods. The intrinsically lower band numbers of FLiP patterns might contribute to slightly lower discriminatory power of this method in comparison to IS*6110 *RFLP. The results indicate that the different number of bands in FLiP and FLAP DNA fingerprints may not necessarily reflect the number of IS*6110* copies in the strains tested. Moreover, the observed differences could be caused by technical difficulties, including mistakes in interpretation of visualized bands and insufficient separation of PCR fragments in poliacrylamide gel, or by difficulties of the PCR amplifying large products itself. It has been shown that PCR-based DNA fingerprinting patterns could result from nonspecific amplification of some of the products [13]. However, the analysis of identically clustered strains clearly showed the highest concordance for the FLiP and IS*6110 *RFLP methods (48 strains, 78,7%). Both FLAP and FLiP methods allowed to group 32 strains (52,4%), whereas the lowest clustering identity was observed for FLAP and IS*6110 *RFLP methods for 23 strains (37,7%).

## 4. Conclusions

In summary, the LM-PCR methods proved to be effective and reproducible for the differentiation of *M. tuberculosis* strains, showing high discriminatory power comparable to that of the IS*6110* RFLP. Based on the previous and present results, the LM-PCR methods seem to be a valuable alternative (highly discriminating, inexpensive, and very fast) to the widely applied and standardized IS*6110* RFLP method. However, the main limitation of the PCR-based methods, including LM-PCR, is the incapability in construction of reference database, especially containing strain patterns of isolates from different laboratories. Therefore, these methods might be rather used as a second-line test for verification of epidemiological links and could be valuable molecular epidemiology tools for analyzing collections with a limited number of strains. Nevertheless, based on our results, it is clear that it is necessary to apply more than one PCR-based method simultaneously.

## Figures and Tables

**Table 1:** The comparison of 61 clustered strains by FLAP, IS*6110*-RFLP, and FLiP analysis.

	Strain	FLAP	IS*6110*-RFLP	FLiP
1	50/8	**F.1**	R.1	**1**
2	674/7		R.2	
3	149/8	**F.2**	R.3	**2**
4	146/7	**F.3**	R.4	**3**
5	319/7	F.4	R.5	
6	19/7			4
7	118/7		R.6	5
8	126/7			
9	147/8	F.5	R.7	6
10	54/8		R.8	7
11	102/7	**F.6**	R.9	
12	176/7			**8**
13	171/8	**F.7**	R.10	
14	412/7			**9**
15	129/7	**F.8**	R.11	
16	41/7			**10**
17	216/8	**F.9**	R.12	
18	307/7			**11**
19	165	**F.10**	R.13	**12**
20	218/8	**F.11**	R.14	**13**
21	230	**F.12**	R.15	
22	9/7			**14**
23	84	**F.13**	R.16	**15**
24	321	F.14	R.17	16
25	65/7	F.15		
26	632			
27	179/8	F.16		
28	386/7			
29	391/7			
30	611			
31	564			
32	152/7	F.17	R.18	17
33	690/7		R.19	18
34	34/7		R.20	19
35	549/7			
36	550			
37	567/7	F.18		
38	696			
39	232/8	**F.19**	R.21	**20**
40	571/7			
41	565	**F.20**	R.22	**21**
42	80/7	**F.21**	R.23	**22**
43	490/7	**F.22**	R.24	**23**
44	704	F.23		24
45	222		R.25	25
46	237/8	**F.24**	R.26	**26**
47	253			
48	1/7	**F.25**	R.27	**27**
49	10/7			
50	601			
51	120/7		R.28	
52	723	F.26	R.29	28
53	91/8		R.30	29
54	459	**F.27**	R.31	**30**
55	306/7	F.28	R.32	31
56	725			
57	724		R.33	
58	108/8		R.34	32
59	671	**F.29**	R.35	**33**
60	305/7	**F.30**	R.36	**34**
61	37/7	**F.31**		**35**

The identical clusters determined in both FLiP and FLAP methods are marked in bold.
